# Controlling the message: preschoolers’ use of information to teach and deceive others

**DOI:** 10.3389/fpsyg.2015.00867

**Published:** 2015-06-23

**Authors:** Marjorie Rhodes, Elizabeth Bonawitz, Patrick Shafto, Annie Chen, Leyla Caglar

**Affiliations:** ^1^Department of Psychology, New York UniversityNew York, NY, USA; ^2^Department of Psychology, Rutgers UniversityNewark, NJ, USA; ^3^Department of Mathematics and Computer Science, Rutgers UniversityNewark, NJ, USA

**Keywords:** evidence selection, teaching, deception, cognitive development, pedagogy

## Abstract

Effective communication entails the strategic presentation of information; good communicators present *representative* information to their listeners—information that is both consistent with the concept being communicated and also unlikely to support another concept a listener might consider. The present study examined whether preschool-age children effectively select information to manipulate others’ semantic knowledge, by testing how children choose information to teach or deceive their listeners. Results indicate that preschoolers indeed effectively select information to meet some specific communicative goals. When asked to teach others, children selected information that effectively spanned the concept of interest and avoided overly restrictive or overly general information; when asked to deceive others, they selected information consistent with the intended deceptive messages under some circumstances. Thus, preschool children possess remarkable abilities to select the best information to manipulate what others believe.

## Introduction

Effective communication often entails the strategic presentation of information: politicians describe uncontroversial portions of their proposals and leave out less palatable details; storytellers present the components of their narratives slowly to build anticipation of major events; teachers present unambiguous examples to help learners obtain new concepts, leaving aside exceptions, and qualifications until the basic ideas are in place. In each case, effective communicators consider the information relevant to the beliefs they wish to communicate, reason about how particular information will shape the mental states of listeners, and present specific information accordingly. That is, good communicators select *representative* information—information that is both consistent with the concept being communicated and also unlikely to support another concept a listener might consider (Griffiths and Tenenbaum, 2001; Shafto and Goodman, 2008). By quite early in development, children can make accurate guesses about the concepts being communicated from representative information (Xu and Tenenbaum, 2007; Gopnik and Wellman, 2012). Less is known, however, about children’s ability to select information in the service of communicating a concept to another.

Here we examine whether preschool-age children make effective use of information to manipulate others’ beliefs by testing whether they strategically select information to teach or deceive their listeners. By quite early in development, children systematically consider their social partners’ mental states when providing information. For example, in the second year of life (ages 18–24 months), infants track whether other people hold true or false beliefs about the locations of objects and intervene by pointing to communicate true locations only when necessary (i.e., only to prevent a person holding a false belief from making a mistake; Knudsen and Liszkowski, 2012a,b; see also Buttelmann et al., 2009). Furthermore, 2-year-olds are more likely to add verbal cues for a partner when pointing alone may produce ambiguity in the referent (O’Neill and Topolovec, 2001), and 3- and 4-year-olds understand the relationship been ambiguous messages and communication failure (Robinson and Robinson, 1982) and produce more informative speech when their partner does not have visual access to a scene (Matthews et al., 2006).

In the later preschool years, children also develop the ability to attend to another’s beliefs in order to effectively select arguments for persuasion. For example, Bartsch et al. (2011) investigated 3, 4–5, and 6–7 year-old children’s persuasion of people and puppets. They found that only the 6–7 year-olds showed greater attention to beliefs for people than puppets. Similarly, Sodian and Schneider (1990) investigated 4–6 year-old children’s abilities to assist or hinder a partner by placing targets in expected or unexpected locations. They found a clear developmental trend progressing from failure to competency over this age range.

In addition to tracking the mental states of others when providing information, children’s early deceptive behaviors also reflect attempts to instill specific mental states in other people. Simple deceptive behaviors, such as denying having performed an action (Lewis et al., 1989), withholding information (Peskin, 1992), or marking an incorrect location (Chandler et al., 1989; Russell et al., 1991; Sodian et al., 1991; Carlson et al., 1998) emerge in the preschool years and are linked to false-belief and inhibitory control (e.g., Talwar and Lee, 2008). These tests of early deceptive behaviors have focused on fairly simple manipulations of episodic knowledge—children deceptively communicating that previous events either did or did not occur. Even in these straightforward contexts, preschool-age children often undermine their own intentions to deceive by accidentally “leaking” information that reveals the truth (Talwar and Lee, 2002). Thus, although prior work has shown that young children attempt to manipulate others’ mental states through deception, based on this work, children’s understanding of the relation between the information they provide and their partners’ mental states appears somewhat precarious.

Here we examine whether preschool-age children can strategically select information to instill particular semantic knowledge in other people. Success on such a task would require selecting the most effective information to communicate particular messages—unlike the tasks described above, which involved a simpler decision of whether to provide information or not.

Previous work examining children’s ability to evaluate the effectiveness of multiple sets of evidence comes primarily from the literature on scientific reasoning, and suggests that metacognitive reasoning about evidence often develops fairly late in childhood (Bindra et al., 1980; Fay and Klahr, 1996; Koslowski, 1996; Chen and Klahr, 1999; Klahr and Chen, 2003; Masnick and Klahr, 2003). For example, preschool-age children often have difficulty deciding whether particular sets of information provide good support for new hypotheses (Rhodes et al., 2008). Indeed, even older children and adults struggle with designing informative interventions in order to generate meaningful evidence (Kuhn et al., 1988; Kuhn, 1989).

Communicative contexts involving simpler concepts might reveal earlier, nascent forms of information-selection abilities, however. For example, Rhodes et al. (2010) found that 6-year-olds select information more strategically when asked to communicate a concept to someone else than when asked to discover a concept for themselves. Thus, communicative contexts may elicit particularly sophisticated use of information. In the present study, we asked preschoolers to choose a representative sample of information to teach or deceive another about a concept, providing a test of whether preschoolers can effectively select information to manipulate the semantic knowledge of other people.

## Materials and Methods

Participants (*N* = 64, 34 female; *M*_age_ = 4.9 years, range = 3.8–6.1 years) were assigned to either Teaching (*N* = 32) or Deception (*N* = 32) conditions. An additional 10 participants began testing but were excluded: three for experimenter error, one for familial interference, and six for not completing the entire study. There were no differences in age across conditions, *F*(3,60) = 1.27, *ns.* All study procedures were approved by the Institutional Review Board at New York University.

**FIGURE 1:**
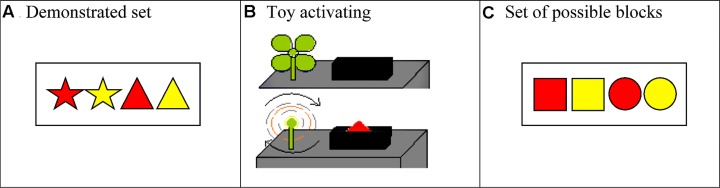
**(A)** Set of four blocks used to demonstrate that all blocks make the toy go. **(B)** The novel toy pictured in the off position and the on position. **(C)** Participants were asked to select two blocks, from this set of four possible blocks, to show Daisy so that she can infer the rule specified by the child’s condition.

Children were introduced to a novel toy and a transparent container filled with blocks. The blocks included four demonstration blocks and four blocks to be used as test (see **Figure 1**), though at the beginning of the experiment, all of the blocks were intermixed in the transparent container. In a seemingly random fashion, the experimenter drew the set of four demonstration blocks from the container and laid them on the table in front of the child in one of two orders, counterbalanced across participants. To familiarize children with the blocks, they were asked to point to each one (e.g., “Can you point to the red triangle?”).

Next children were shown a demonstration of how the toy worked. Half of children within each condition were taught that placing any of the blocks on the machine would “make it go” (cause an attached propeller to spin), whereas the other half were taught that only red blocks would “make it go^1^.”

For example, to teach that all blocks could turn on the toy, children were told, “Now we’re going to play a game with my special toy. This toy is special because my blocks make it go. All of my blocks make it go! Let me show you how it works.” The experimenter proceeded to place each of the blocks on top of the machine. Each time the experimenter said, “Oh look! Did you see the toy go? This block made my toy go.” The propeller turned on each time. After all four demonstrations, the experimenter picked up each block one at a time and asked, “So did this block make the toy go?” All of the participants answered correctly. The experimenter reiterated that all of the blocks activated the machine, including the rest of the blocks (the test set) in the transparent bucket, by saying, “All of the blocks make it go! All of the blocks we have laid out here, and All of the blocks in the bucket too!” The experimenter then put the demonstration blocks back in the transparent container.

The procedure for children assigned to learn that only red blocks activated the toy was similar, but modified as necessary (e.g., “Let’s put this yellow triangle on the toy and see what happens. [Toy does not activate]. Oh look- the toy didn’t go! The yellow triangle does not make my toy go”).

Children were then introduced to a puppet, “Daisy,” and told that she did not know which blocks would activate the toy. The puppet was then removed from sight. In the Teaching condition, children were then told that their goal was to teach Daisy how the toy worked. For children who had been told that all blocks made the toy go, the experimenter said, “A little while ago, I had a different toy that looked just like this toy, but for my old toy, only red blocks made it go.” They were reminded that for the current toy *all* the blocks make it go and told that their goal was to help Daisy learn that all blocks make it go. For children who were told that only red blocks make the toy go, they were told, “Daisy might be wrong about how the toy works and think all blocks make it go.” They were reminded that really only red blocks make it go.

In the Deception condition, children who had been told that really all blocks activate the toy were told, “Let’s play a fun trick on Daisy and make her think that only red blocks make it go.” They were then reminded that in reality, *all* the blocks make it go, but that their goal was to trick Daisy to make her think that only red blocks make it go. Children who had been told that really only red blocks activate the toy were told, “Let’s play a fun trick on Daisy, and make her think that all blocks make it go.” They were then reminded that in reality, *only* red blocks make it go.

Children in all conditions were then presented with four new blocks (**Figure 1**)—the set of possible information—and were asked to select two of the four blocks to communicate the intended concept to Daisy (e.g., “Let’s pick the best two blocks to show her” [Teaching: “so she will learn that (all blocks/only red blocks) make it go”; Deception: “to trick her into thinking that (all blocks/only red blocks) make it go”]). In all conditions, the experimenter asked, “So remind me one more time. How many blocks are we going to show Daisy?” Corrective feedback was given when necessary. Daisy was then brought back into view, and the experimenter said, “Remember, you can pick any of these four blocks to show Daisy to help her think about how the toy works.” Children were then asked to select two blocks. Daisy was then put away and the experimenter asked, “Remind me, what really makes the toy go?” The majority of children in both conditions correctly generated the response consistent with the rule they had been taught (“all blocks” or “red blocks”; Teaching, 26/32; Deception, 28/32).

## Results

**FIGURE 2:**
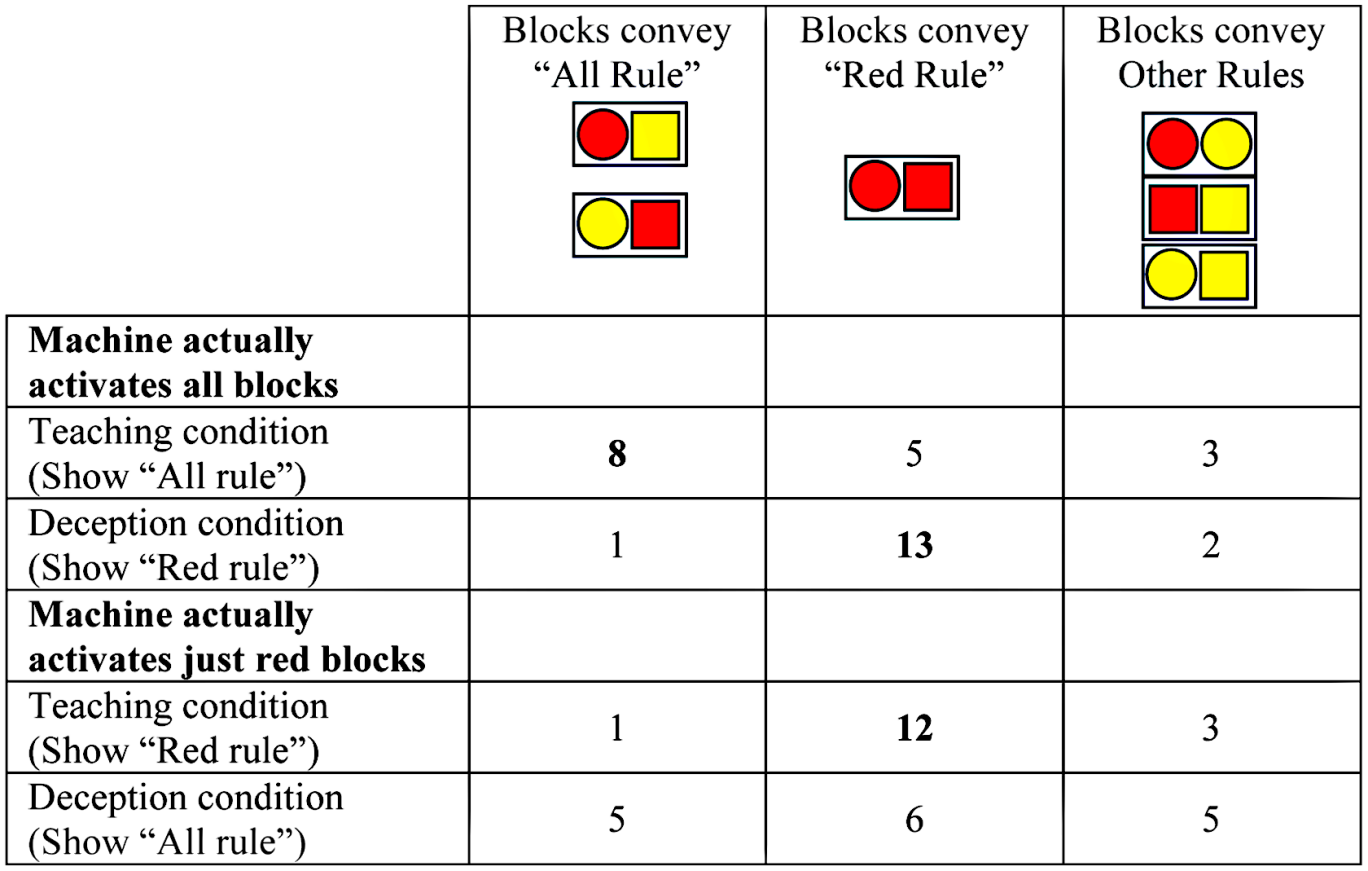
**Number of children choosing blocks in the Teaching and Deception conditions.** Two possible block pairings convey the “All Rule”; one pair conveys the “Red Rule”; three remaining pairs convey other rules. (Probability of randomly selecting blocks consistent with “All Rule” = 2/6, “Red Rule” = 1/6, “Other Rules” = 3/6).

Children’s information selection uniquely and unambiguously fell into one of three categories: teaching, deception target, other (**Figure 2**). Children effectively selected block pairs to communicate the belief specified by their condition; their selections differed depending on whether they were given a teaching goal or a deceptive goal, χ^2^(2, *N* = 64) = 12.34, *p* = 0.002. Overall, when children were asked to teach, 20 picked the best set of information to communicate the truth, six picked block pairs consistent with the deception target, and six picked another set of blocks. When children were asked to deceive, 18 picked the best set of blocks to deceive, seven picked the best set to communicate the truth, and seven picked another set. Children’s response patterns reliably differed from chance^2^ in both Teaching and Deception conditions (by binomial tests: Teaching, *p* < 0.001; Deception, *p* < 0.01).

Within the Teaching condition, there was no effect of whether children were asked to teach the “all rule” or “red rule,” χ^2^(2, *N* = 32) = 2.13, *ns*; children’s responses in both conditions were significantly different than the predicted distribution if responses were at chance [see **Figure 1**; “all rule”: χ^2^(2, *N* = 16) = 6.50, *p* = 0.04; “red rule”: χ^2^(2, *N* = 16) = 39.31, *p* < 0.001]. In the Deception condition, however, children’s responses differed by the deceptive message, χ^2^(2, *N* = 32) = 8.42, *p* = 0.02. When children were asked to deceive the puppet into thinking that only red blocks operated the toy (when really all blocks did so), children reliably picked the set of blocks that communicated this message (binomial, *p* < 0.001) and were significantly different than the predicted distribution if responses were at chance, χ^2^(2, *N* = 16) = 46.06, *p* < 0.001. In contrast when children were asked to deceive the puppet into thinking that all blocks turned on the toy (when really only red blocks did so), children did not select the correct response above chance levels and children’s responses did not differ from the predicted distribution if responses were at chance, χ^2^(2, *N* = 16) = 5.31, *p* = 0.07; see **Figure 1**.

Half of children (*n* = 32, the second half tested in both conditions) were asked at the end of the session whether, after seeing the chosen blocks, the puppet would understand how the toy works or whether she would make mistakes. Responses to this question varied by condition as expected, χ^2^(1, *N* = 32) = 9.34, *p* = 0.002; most children (12/16) in the Teaching condition expected her to know how the toy works, whereas most children (12/16) in the Deception condition expected her to make mistakes. That is, critically, children understood that in the Deception condition they were conveying incorrect information that would lead the puppet to make mistakes.

Across conditions, children’s responses to the final question were also associated with whether they showed the best information to present the truth (of children who presented the best set of blocks to communicate the truth, 12 said she would know how the toy works and seven said she would make mistakes; of children who present any other block pairs, nine said she would make mistakes, and four said she would know how the toy works, χ^2^(1, *N* = 32) = 3.90, *p* < 0.05. These results also support the claim that children understand the relationship between the information presented and the effect of this information on another’s understanding. Whether children picked the best information as specified by their condition, however, (e.g., the best set of blocks to communicate the truth in the Teaching condition but to communicate the intended deceptive message in the Deception condition), was not related to whether they answered this question accurately (*p* = 0.69).

## Discussion

In this study, preschoolers effectively selected information to teach or deceive other people. Both conditions require a level of strategic information selection that goes beyond what has been previously demonstrated by children of this age. When children were asked to teach that *all* blocks operate the machine, all of the block pairs that children could choose were consistent with the truth (i.e., that all blocks turn on the toy). Yet children were more likely to select information that spanned the concept and thus avoided communicating an overly restricted rule (e.g., that only squares or yellow blocks turn on the toy). That is, while it would have been technically accurate for children to select two red blocks, two yellow blocks, two circle blocks, or two square blocks, only the two possible response pairs that spanned both concepts (a red square and yellow circle, or a yellow square and red circle) were counted as accurate in the “all rule” condition. Children reliably selected these concept-spanning pairs. In contrast, when children were asked to teach a narrower rule (i.e., that only red blocks activate the toy), they reliably selected blocks to communicate this concept.

In the Deception condition, when children were told that really all blocks activate the toy but asked to “trick” the puppet into a believing that only red blocks did so, children were able to inhibit their knowledge of the true rule and effectively select information that would communicate the false, overly restricted rule. In contrast, when children were told that only red blocks activated the toy and were asked to trick the puppet into believing instead that all blocks did so, they failed to do so at statistically significant rates. One thing to note is that this is the only condition where successful communication of the intended message would have required the presentation of *false* information—telling the puppet, for example, that a block would activate the toy when it really wouldn’t do so (the other Deception condition simply required children to present a misleading set of accurate but too restrictive information). Thus, children might not have reliably communicated the intended message in this condition because they were either unwilling to present information that was explicitly false, or did not understand that doing so was an option. Alternately (or additionally), they could have had trouble selecting false information because they could not inhibit their own knowledge of the true rule. Finally, children may have been more likely to succeed in the “red blocks” condition because the rule “red blocks” helped provide information about which blocks (the red ones) that would be optimal for selection. Examining why children did not reliably present false information here, when they were willing to present accurate but deceptive information, thus remains an important question for future work.

In our study, children’s ages spanned older 3-year-olds up to 6-year-olds. Although most children were 4- to 5-year-olds, one might wonder whether older children in our conditions were more likely to succeed at our task than younger ones. For example, children’s abilities to select persuasive arguments (Bartsch et al., 2011) and effectively cue or deceive others on target-search tasks (Sodian and Schneider, 1990) improve across the later preschool and early elementary school years. Yet, we did not find any correlation between age and success on our task, and splitting our sample of 64 children in half by age also revealed no difference between the younger half of 32 (18 succeeding) and the older half (13 succeeding), χ^2^(2, *N* = 64) = 0.26, *ns*. Future work might investigate the degree to which even younger children succeed at information selection tasks like the one used here, and also why developmental differences across early childhood arise for some information selection tasks but not for others.

In future work, it will also be useful to examine in more detail why children can effectively select sets of information in some communicative contexts when they have trouble doing so in some tests of their scientific reasoning (Bindra et al., 1980; Fay and Klahr, 1996; Koslowski, 1996; Chen and Klahr, 1999; Klahr and Chen, 2003; Masnick and Klahr, 2003). One possibility is that different reasoning mechanisms support information communication vs. information discovery (Rhodes et al., 2010). Yet, another possibility is that children succeeded in the present task because it involved simpler concepts than have been tested in prior work. Indeed, Ruffman et al. (1993) and Koerber et al. (2005) found that the age at which children can successfully reason about how patterns of evidence lead to particular mental during scientific reasoning depends on features of the task complexity. Similarly, in our on-going work, we have found that even in instances of concept communication, preschool-age children show less systematic information selection when the number of dimensions that varies across the sets of information increases (and thus children have to consider a much larger hypothesis space). These differences in task complexity may also explain why other research that examines children’s use of information to persuade or deceive others (e.g., Sodian and Schneider, 1990; Bartsch et al., 2011) shows proficiencies later in development than found here. Systematically comparing children’s information selection across different types of learning contexts for tasks equated for these stimulus features is thus necessary to determine the boundaries and developmental timescale of children’s abilities.

The present study extends prior work on the development of theory of mind (Knudsen and Liszkowski, 2012a,b) and deception by showing that not only can children consider their social partner’s current and intended mental states to provide information about whether a prior event occurred, they can strategically select between multiple sets of truthful information to instill specific semantic knowledge in other people. These results contribute to a growing body of evidence that, from an early age, children exhibit surprising, seemingly sophisticated abilities to learn in and reason about social and communicative contexts.

## Conflict of Interest Statement

The authors declare that the research was conducted in the absence of any commercial or financial relationships that could be construed as a potential conflict of interest.

## Footnotes

1 Data collection proceeded in two phases. First, 32 children participated (randomly assigned to Teaching or Deception conditions) in the procedure in which children were told that *all blocks* make the toy go. Second, another 32 children participated (randomly assigned to Teaching or Deception conditions) in the procedure in which children were told that *only red blocks* make the toy go. There were no differences in age across these samples, *t*(62) = 0.63, *p* = 0.531. Of the children who participated in the first batch of data collection, half first participated in another study (participating in the other study did not affect the present results; χ^2^(4) = 6.12, *p* = 0.2).

2 We used the most conservative chance comparison of 1/3; for the red demonstrations, chance is smaller (1/6).
